# Supplementary material for: Pro12Ala PPAR-γ2 and +294T/C PPAR-δ Polymorphisms and Association with Metabolic Traits in Teenagers from Northern Mexico
Source: Genes (Basel). 2020 Jul 10;11(7):776. doi: 10.3390/genes11070776 (PMC7397260; doi:10.3390/genes11070776)
Supplement: Supplementary file 1 [file genes-11-00776-s001.pdf]

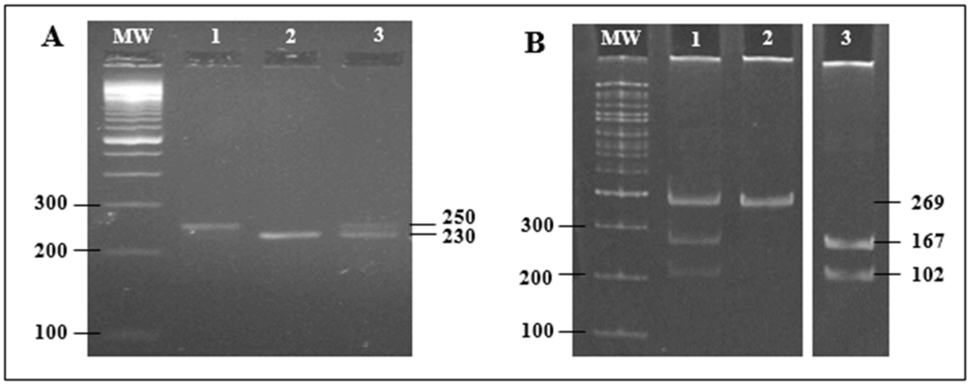

Figure S1. A) Pro12Ala PPAR-**γ2** PCR genotypes. MW 100 bp marker ladder; lane: 1 GG genotype (250 bp); lane: 2 CC genotype (230 bp); lane 3: GC genotype (250 and 230 bp). **B)** +294T/C PPAR-**δ** RFLP genotypes. MW 50 bp marker ladder; lane 1: TC genotype (269, 167 and 102 bp); lane 2: TT genotype (269 bp); lane 3: CC genotype (167 and 102 bp).
